# Transcriptomic and Metabolomic Studies Disclose Key Metabolism Pathways Contributing to Well-maintained Photosynthesis under the Drought and the Consequent Drought-Tolerance in Rice

**DOI:** 10.3389/fpls.2016.01886

**Published:** 2016-12-21

**Authors:** Xiaosong Ma, Hui Xia, Yunhua Liu, Haibin Wei, Xiaoguo Zheng, Congzhi Song, Liang Chen, Hongyan Liu, Lijun Luo

**Affiliations:** ^1^Shanghai Agrobiological Gene CenterShanghai, China; ^2^College of Plant Sciences and Technology, Huazhong Agricultural UniversityWuhan, China

**Keywords:** drought-tolerance, RNA-sequencing, metabolome, osmotic adjustment, antioxidant capacity, photosynthesis

## Abstract

In contrast to wild species, drought-tolerance in crops requires a fully functional metabolism during drought (particularly photosynthetic processes). However, the link between drought-tolerance, photosynthetic regulation during drought, and the associated transcript and metabolic foundation, remains largely unknown. For this study, we used two rice cultivars with contrasting drought-tolerance (the drought-intolerant cultivar IRAT109 and the drought-tolerant cultivar IAC1246) to explore transcript and metabolic responses to long-term drought. The drought-tolerant cultivar represented higher osmotic adjustment and antioxidant capacity, as well as higher relative photosynthesis rate under a progressive drought stress occurred in a modified field with shallow soil-layers. A total of 4059 and 2677 differentially expressed genes (DEGs) were identified in IRAT109 and IAC1246 between the drought and well-watered conditions, respectively. A total of 69 and 47 differential metabolites (DMs) were identified between the two treatments in IRAT109 and IAC1246, respectively. Compared to IRAT109, the DEGs of IAC1246 displayed enhanced regulatory amplitude during drought. We found significant correlations between DEGs and the osmolality and total antioxidant capacity (AOC) of both cultivars. During the early stages of drought, we detected up-regulation of DEGs in IAC1246 related to photosynthesis, in accordance with its higher relative photosynthesis rate. The contents of six differential metabolites were correlated with the osmotic potential and AOC. Moreover, they were differently regulated between the two cultivars. Particularly, up-regulations of 4-hydroxycinnamic acid and ferulic acid were consistent with the performance of photosynthesis-related DEGs at the early stages of drought in IAC1246. Therefore, 4-hydroxycinnamic acid and ferulic acid were considered as key metabolites for rice drought-tolerance. DEGs involved in pathways of these metabolites are expected to be good candidate genes to improve drought-tolerance. In conclusion, well-maintained photosynthesis under drought should contribute to improved drought-tolerance in rice. Metabolites play vital roles in protecting photosynthesis under dehydration *via* osmotic adjustments and/or antioxidant mechanisms. A metabolite-based method was thus an effective way to explore drought candidate genes. Metabolic accompanied by transcript responses to drought stress should be further studied to find more useful metabolites, pathways, and genes.

## Introduction

With increased global water shortage, enhancing drought-resistance in cereal crops breeding is one of the most important goals (Sivamani et al., 2000; Luo, 2010; Hu and Xiong, 2014). Drought-resistance in crops is mostly determined by the relative yield under stressed conditions compared to the yield under normal conditions (Yue et al., 2006; Luo, 2010). Two aspects are important: drought avoidance and drought tolerance (Luo, 2010; Hu and Xiong, 2014). Drought avoidance depends on the development of a large and deep root system to uptake water from the soil and reducing transpiration *via* stomata closure. Drought tolerance mainly results from osmotic adjustment, protective mechanisms or scavenging of reactive oxygen species (ROS), enabling unhampered life processes (e.g., photosynthesis, growth, development) and to preserve membranes during dehydration stress (Blum, 2005; Luo, 2010; Ning et al., 2010; Du et al., 2012; Jeong et al., 2012; Uga et al., 2013; You et al., 2013; Hu and Xiong, 2014). A plant with improved drought-avoidance might utilize more water *via* a well-developed root system, while a plant with improved drought-tolerance maintains its basic life activities even when dehydrated (Blum, 2005; Luo, 2010). Drought-avoidance *via* enlargement of the root system plays a primary role in plant responses to drought and the molecular basis for this has been studied and applied in the breeding of drought-resistant cultivars (Pietta, 2000; Uga et al., 2013; Lou et al., 2015). Drought-tolerance plays an important role during the later stage of drought and has great potential to enhance drought-resistance. However, due to their complexity, the molecular mechanisms of drought-tolerance remain largely unknown (Hu and Xiong, 2014).

Plants have various acclimation responses that enable survival under drought. These acclimation responses include stomata closure, leaf rolling, and inhibition of growth and development (Yordanov et al., 2000). However, drought-resistance of a crop needs to result in a relatively higher yield under drought, rather than mere survival. It is therefore important for a crop to maintain its normal metabolic process as long as possible during drought stress (Luo, 2010; Hu and Xiong, 2014). In this aspect and particularly for crops, drought acclimation differs from drought resistance.

Photosynthesis is the most important metabolic process for carbon assimilation in plants. Down-regulation of photosynthesis is the primary acclimation response to drought stress (Chaves et al., 2009; Saibo et al., 2009). Down-regulation of photosynthesis could be due to a decrease of available CO_2_ caused by stomata closure or the subsequent photo-oxidative damage induced by an accumulation of ROS under dehydration (Chaves et al., 2009; Saibo et al., 2009). However, many mechanisms of drought-tolerance, such as osmotic adjustment *via* osmolytes (e.g., proline, betaine, glycine), protective metabolites (e.g., trehalose, raffinose, mannitol), proteins (e.g., chaperone and dehydrin), and ROS scavenging systems (e.g., superoxide dismutase, peroxidase, and ascorbic acid) play roles in maintaining normal photosynthesis under dehydration, resulting in relative higher biomass accumulation and yield (Serraj and Sinclair, 2002; Valliyodan and Nguyen, 2006; Saibo et al., 2009; Hu and Xiong, 2014). Therefore, maintained photosynthetic capacity under drought is an important feature of drought-resistance in crops, in which metabolites may play an important role. However, expression of genes that are related to photosynthesis during drought stress, metabolites involved in the protection of photosynthesis, and their associations with drought-tolerance have not been intensively studied (Chaves et al., 2009).

Recently, metabolic responses to drought stress in plants have attracted more attention, as many metabolites are considered to play central roles in stress-tolerances (Garg et al., 2002; Taji et al., 2002; Nuccio et al., 2015). A metabolite is a better target for improving drought-tolerances than a single gene as it always resulted from interactions of various genes and pathways, resulting in systemic effects in responses to stress (Garg et al., 2002; Taji et al., 2002; Nuccio et al., 2015). For example, trehalose was reported to be associated with drought-tolerance (Garg et al., 2002; Nuccio et al., 2015). Over-expression of *trehalose-6-phosphate phosphatase*, a key enzyme gene of the trehalose metabolism, increased trehalose accumulation in rice and maize, and consequently enhanced their drought-tolerance significantly, as well as their performance under well-watered conditions *via* regulating photosynthesis (Garg et al., 2002; Nuccio et al., 2015).

Rice is one of the most important cereal crops, feeding >50% of the global population. However, rice production requires large amounts of water and is extremely susceptible to drought stress (Zhang, 2007; Luo, 2010; Hu and Xiong, 2014). Although a large number of drought-resistance genes have been identified (Hu et al., 2006; Ning et al., 2010; Zhu and Xiong, 2013; Hu and Xiong, 2014; Fang et al., 2015; Jung et al., 2015), the molecular basis of drought-tolerance remains largely unknown. It is therefore essential to distinguish the mechanisms of drought-tolerance from those of drought-avoidance. Unfortunately, it is difficult to separate drought-tolerance and drought-avoidance in normal field conditions as the intensity of drought stress varies significantly even among neighboring plants due to differences in the depth of their root systems (Yoshimura et al., 2008; Parent et al., 2010). Instead, experiments with plants in pots are regularly applied to study drought-tolerance. A volume limited pot prevents roots to access water from deeper levels (Parent et al., 2010). However, this method has its own limitations, as the water content needs to be individually controlled in each pot. When large numbers of pots are involved, the precise control of the water content in each pot becomes very difficult, leading to unwanted variation in the intensity of drought stress among pots. To solve this problem, we designed our experiment using a modified field with limited soil layer depth. In this experimental field, roots of different rice cultivars were neutralized at equivalent depth just like in a pot. Additionally, the soil-water content in this small field could be well-homogenized to avoid the intensity variations of drought stress among rice cultivars.

Methodologies of systems biology, such as transcriptomics and metabolomics are powerful tools to explain complex traits, such as drought resistance (Zheng et al., 2010; Wang et al., 2011; Prince et al., 2015), salt tolerance (Mizuno et al., 2010; Zhao et al., 2014), tolerance to low and high temperature stress (Caldana et al., 2011), and grain quality (Venu et al., 2011). Many important functional genes and gene networks related to these complex traits have been effectively explored (Sasidharan et al., 2013; Secco et al., 2013; Avramova et al., 2015). Different from the capacity of water uptake *via* roots, drought-tolerance reflects the level of life processes in leaf samples under drought stress. To explore the molecular mechanisms of drought-tolerance, we studied the transcript and metabolism of two rice cultivars with contrasting drought-tolerances by subjecting them to a long-term drought in our modified field experiment. We address the following questions: (1) How will osmotic adjustment, antioxidant mechanisms, and photosynthesis respond to the progressive drought in rice cultivars of contrasting drought-tolerances? (2) How will transcripts and metabolic pathways respond to the progressive drought in rice cultivars of contrasting drought-tolerances? (3) Are there any featured metabolites in rice tolerance to drought? (4) How are these candidate metabolites and their related genes enhancing drought-tolerance?

## Materials and methods

### Plant materials

Two rice cultivars of contrasting drought-tolerance were used in this study. Although both IRAT109 and IAC1246 are drought-resistant cultivars, IRAT109 has a low drought-tolerance, while IAC1246 has a high drought-tolerance as pre-evaluated in a pot experiment in 2011 (Table S1). They both belong to *japonica* subspecies and have been widely used in water-saving and drought resistance rice (WDR) breeding (Luo, 2010).

### Field experiment and sampling strategies

The field experiment was conducted in the drought-resistance facility at Baihe Experimental Station in 2014. The canopy of the facility was normally opened, and could be closed on rainy days to enable continuous drought. IRAT109 and IAC1246 have distinctly different root lengths, leading to varied capacities for water access. Informed by previous experiments that used pots with 30 cm soil depth and enabled a good separation of drought-tolerance and drought-avoidance, the depth of the soil layer in the experimental field was also limited to 30 cm. With this design, the development of roots below this depth was equal between both cultivars and therefore the differences in drought-avoidance could be largely mitigated. Moreover, planting rice in the experimental field condition rather than in pots led to more homogenous levels of soil-water content.

Plants were cultured in a plot of 10 rows × 10 hills with three replicates each for drought-stressed and well-watered conditions. The interval between hills was 18 cm. Individual seedlings were transplanted into each hill on the 20th of June, about 30 days after germination. We started the drought treatment on the 16th of July and continued the artificial drought for 38 days until re-watering on the afternoon of the 22th of August. The field arrangement was followed with the single factor randomized block design.

**Figure 1:**
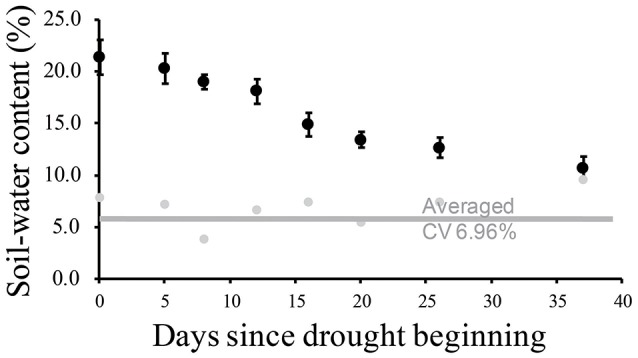
**Soil water contents monitored during drought treatment and related coefficients of variance**.

Physiological traits were measured using three replicates of leaf samples. Each replicate contained the three top leaves of the main tillers from each plot. A further three replicates of leaf samples were collected for transcript and metabolite identification. These leaf samples were immediately frozen in liquid nitrogen until they were used for further experiments. We measured the soil water content once every 3–5 days at each plot in the drought-stressed field to monitor and categorize the degree of drought stress at a depth of 30 cm (Figure 1). We harvested the leaf samples at six time points: 24th of July (A, later tillering stage), 29th of July (B, booting stage), 5th of August (C, booting stage), 11th of August (D, heading for IRAT109), 22nd of August (E, heading for IAC1246), and 23th of August (F, re-watering) (Table S2). Judging from the soil-water contents, we categorized the time points A and B as mild drought periods (I), time points C, D, and E as severe drought periods (II), and time point F as re-watering stage (III). All leaf samples were strictly collected between 13:00 and 14:00 at each time point.

### Measurement of agronomic and physiological traits

Relative water content (RWC) and osmolality were measured to reflect the water status and osmotic adjustment of the leaf samples. We estimated RWC as: (fresh weight - dry weight)/(saturate weight - dry weight). We conducted the measurement of the osmolality via the Vapro™ vapor pressure osmometer (Wescor Model 5600). We measured the total antioxidant capacity (AOC), which reflects the capacity for ROS scavenging, using the total antioxidant capacity assay kit (Nanjing Jiancheng Bioengineering Institute, Jiangsu, China). The proportion of dead leaf was observed and estimated after re-watering on the 23th of August. We measured biomass and grain yield of both rice cultivars after harvest and calculated the drought-tolerance coefficient as: grain yield in drought-stressed field divided by grain yield in well-watered fields. Photosynthesis rates of IRAT109 and IAC1246 under both conditions were measured in 2016. Seeds of IAC1246 and IRAT109 were germinated on the 25th of May. On the 30th of Jun, two seedlings of each IRAT109 and IAC1246 were transplanted into a plastic pot (30 × 20 × 40 cm) with 30 cm soil depth. Three pots were never irrigated from 20th of July onwards and served as drought treatment. A further three pots were kept well-watered and used as controls. Photosynthesis rate was measured using the LCPRO+Photosynthesis System (ADC BioScientific Ltd.) 3, 7, 11, and 15 days following drought (DAT) (between 9:30 a.m. and 10:30 a.m.), respectively. The soil water content was also measured. We applied independent *t*-tests to detect any significant differences of measured physiological traits between treatments at each time point. We used two-way ANOVA to analyze the impact of different cultivars and drought stress on yield and biomass. All statistical analyses were conducted with the software SPSS 15.0.

### Procedures of RNA-Seq libraries and mapping reads to the reference genome

We extracted the total RNA using the PureLink® Plant RNA Reagent (Life Technologies). We used the qualified RNA samples for library construction following the specifications of the TruSeq® RNA Sample Preparation v2 Guide (Illumina) and conducted the RNA-seq with Illumina Hiseq 2500 in Shanghai Majorbio Biopharm Technology Co., Ltd. (Shanghai, China). We used SeqPrep to strip adaptors and/or merging paired reads with overlap into single reads (https://github.com/jstjohn/SeqPrep) and used Sickle to remove low-quality reads (https://github.com/najoshi/sickle). We then assembled the clean data using the software Cufflinks and mapped them to the reference genome (Nipponbare, msu7.0) with mitochondrial and chloroplast genomes (http://rice.plantbiology.msu.edu/) *via* Tophat with no more than two base mismatches allowed in the alignment (Trapnell et al., 2012). The basic information of the RNA-sequencing data is provided in Table S3.

### Quantification of gene expressions and identification of differentially expressed genes

We determined the gene expression levels with the Fragment Per Kilobase of exon per Million fragments mapped (FPKM) method via the widely applied software Cuffdiff (Trapnell et al., 2012). We validated the measured levels of gene expression *via* the qPCR method, as previously described by Xu et al. (2015). Nine genes, including known drought-resistance genes and several candidate genes were selected for validation (Table S4).

We investigated the differentially expressed genes (DEGs) for both drought-stressed and well-watered conditions from the stage of later tillering (time point A) to the booting stage for IRAT109 and IAC1246, respectively. Since IRAT109 has biological replicates, we determined its DEGs *via* a false discovery rate (FDR) < 0.05 and a logarithm two-fold change |log_2_FC| ≥ 1. Given the mixed nature of the cDNA library of IAC1246, we determined its DEGs with a *p* < 0.05 and |log_2_FC| ≥ 1. Plants exposed to drought commonly delay their development and we observed this as well in our experiment. Therefore, some of the differences in terms of transcripts between samples from the well-watered and drought-stressed conditions should be a result of delayed development. If a gene was determined as a DEG between any two time points under the well-watered condition, it could be considered as development-dependent gene. To avoid any unsymmetrical impacts due to the delayed development, the development-dependent gene was excluded from the final database as its difference in expressions between drought and well-watered conditions could be caused by the difference in development.

### Enrichment analyses of gene ontology and KEGG pathways and integrated pathway analysis via mapman

We conducted enrichment analyses of Gene Ontology (GO) and KEGG pathways based on the DEGs of each drought period in IRAT109 and IAC1246, respectively. Enrichment analysis of the KEGG pathway and Gene Ontology (GO) were conducted using the total DEGs that were detected during different periods of the drought. Time-series analyses were conducted for the energy-, osmolality-, and antioxidant-related DEGs based on the fold change value of gene expression, which was calculated as log_2_[(FPKM-D+0.00001)/(FPKM-W+0.00001)]. If the fold change values exceeded a range from −6 to 6, they were treated as −6 or 6 in the heatmap. The definition for energy-related DEGs is: DEGs involved in KEGG pathways of photosynthesis-antenna proteins (ko00196), carbon fixation in photosynthetic organisms (ko00710), carbon fixation pathways in prokaryotes (ko00720), photosynthesis (ko00195), and oxidative phosphorylation (ko00190). The definition of osmolality and antioxidant-related DEGs is: DEGs significantly correlated with osmolality and T-AOC (using Pearson's coefficient). MapMan 3.6.0RC1 (http://mapman.gabipd.org/web/guest/mapman) was utilized to visualize altered pathways that were involved in drought-responses at each sampling time point (A–F), stage, or the whole drought period (A–E, using the median log_2_FC values of the five stages for each DEG) (Thimm et al., 2004).

### Sample preparation for GC/MS

60 mg of samples were put in 1.5 ml centrifuge tubes and mixed with 360 μL methanol and 40 μL internal standard containing 0.3 mg/mL L-2- chloro–phenylalanine. We ground the samples with a Tissuelyser-48 (60 Hz, 2 min) and extracted all homogenates *via* ultrasonic extraction for 30 min. Subsequently, we added chloroform (200 μL) and distilled water (400 μL), and conducted a final ultrasonic extraction for 30 min. We centrifuged the extracts at 14000 rpm, at 4°C, for 10 min, then transferred 700 μL of each of the supernatants into a glass vial, and evaporated all fluid with a quick spin concentrator. We added 80 μL methoxyamine hydrochloride dissolved in pure pyridine (15 mg/mL) to each glass vial, vortexed for 2 min and then placed the mix into a shaker-incubator (37°C) to allow oxidation for 90 min. The samples were then taken out and 80 μL of BSTFA (containing 1% TMCS) derived reagents and 20 μL of n-hexane were added to each, vortexed for 2 min, and the reactions were allowed to proceed for 60 min at 70°C. We then left the samples at room temperature for 30 min and used them for our GC/MS analysis.

### Procedures of GC/MS analysis

We injected 1 μL aliquots of each of the derivatized solutions in split less mode into the Agilent 7890A-5975C GC-MS system (Agilent, USA). Separation was carried out on a non-polar DB-5 capillary column (30 m × 250 μm I.D., J&W Scientific, Folsom, CA) with high purity helium as carrier gas at a constant flow rate of 1.0 mL/min. The GC temperature programming began at 60°C, continued with 8°C/min oven temperature ramps to 125°C, 4°C/min to 210°C, 5°C/min to 270°C, 10°C/min to 305°C, and a final 3 min maintenance at 305°C. We held the electron impact (EI) ion source at 260°C with a filament bias of–70 V. We used full scan mode (m/z 50–600) with an acquisition rate of 20 spectrum/second in the MS setting.

### Data analysis and validation for metabolites

We analyzed the acquired MS data from GC/MS with the ChromaTOF software (v 4.34, LECO, St Joseph, MI). We normalized the data set using the sum intensity of the peaks in each sample. The data sets resulting from GC-MS were separately imported into the SIMCA-P+ 14.0 software package (Umetrics, Umeå, Sweden). Principal component analysis (PCA) and (orthogonal) partial least-squares-discriminant analysis [(O) PLS-DA] were carried out to visualize the metabolic alterations among experimental groups after mean centering and unit variance scaling. We used variable importance in the projection (VIP) to rank the overall contribution of each variable to the (O)PLS-DA model, and considered variables with VIP > 1.0 as relevant for group discrimination. We based the identification of detected metabolites on the available reference standards in the lab of Shanghai Bo-yuan Biotechnology Co., Ltd (Shanghai, China), the NIST 11 standard mass spectral databases and the Feihn databases linked to the ChromaTOF software. The signal with a similarity of >70% was considered as the reference standard.

To avoid possible bias from the detection of metabolites between the two cultivars, we only involved the metabolites detectable in both IRAT109 and IAC1246 in all further analyses. We determined metabolites with both multivariate and univariate statistical significance (VIP > 1.0 and *p* < 0.05) as differential metabolites. Principal component analysis was conducted to visualize the impact of the drought on the rice metabolome. The annotations of osmolality and antioxidant-related metabolites were based on the correlation analysis conducted between the contents of metabolites and physiological traits using Pearson's coefficient (|r| > 0.6, *p* < 0.05). Correlations between differential metabolites and DEGs were conducted using Pearson's coefficient (|r| > 0.6, *p* < 0.05).

Rarely detected in plants of previous studies, D-Arabitol and ribitol (also named adonitol) were validated in IAC1246 leaf samples (collected in 2016) *via* GC/MS (7890B-5977A, Agilent, USA) at Shanghai Luming Biotechnology Co., Ltd (Figure S1). D-(+)-Arabitol (A3381, Sigma-Aldrich Ltd.) and adonitol (A5502, Sigma-Aldrich Ltd.) were used as standards for validation.

## Results

### Drought-tolerance and related physiological traits estimated for IRAT109 and IAC1246

During drought treatment, the soil water content was maintained above 18.0% for the first 12 days (covering A and B time points) and was decreased to less than 15.0% thereafter (covering from C to E time points) (Figure 1). Based on the decrease of the soil-water content, we categorized time points A and B as mild drought periods I, while we categorized time points C, D, and E as severe drought periods II. The range of the coefficient of variation of the soil water content was an average of 6.96% (Figure 1), indicating homogenous levels of soil-water content.

**Figure 2:**
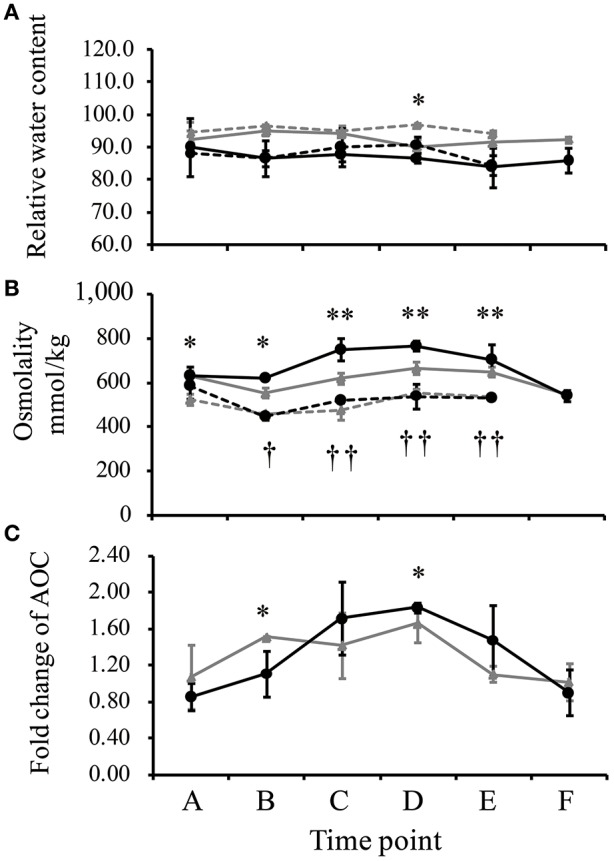
**Relative water content of leafs (RWC, %) (A)**, osmolality **(B)**, and regulation of total antioxidant capacity (AOC) **(C)** measured during drought treatment (time points A–E) and after re-watering (time point F). The regulation of AOC is depicted as fold-change during drought. The black line represents IAC1246, the gray line represents IRAT109, the solid line represents drought stress, and the dotted line represents normal conditions. “^*^” and “^**^” indicate significances in IRAT109 at *p* < 0.05 and *p* < 0.01, respectively. “^†^” and “^† †^” were significant for IAC1246 at *p* < 0.05 and *p* < 0.01, respectively.

The leaf relative water content (RWC) of IRAT109 ranged from 94.2 to 97.1% in the well-watered field and slightly decreased to a minimum of 90.2% in the drought stressed field (Figure 2A). Similarly, we only detected a limited decrease of the leaf RWC for IAC1246 in the drought-stressed field (ranging from 83.9 to 90.2%), compared to those in the well-watered field (ranging from 84.5 to 90.1%). Significant differences of leaf RWC between well-watered and drought treatments were only detected at time point D in IRAT109 (Figure 2A). Although RWC was maintained at normal levels throughout the drought treatment, the osmolality was significantly increased after time point C and subsequently decreased after re-watering (Figure 2B). It is worth noting, that levels of the osmolality in IAC1246 from the time point C to E were significantly higher than those of IRAT109 (Figure 2B), while their levels in the well-watered treatment were equivalent. The AOC of IRAT109 was found to start increasing at time point B and its fold changes stayed at a moderate level from time points C to D. We then found its fold-change (drought/well-watered) to decrease to nearly 1 at time point E although drought stress still prevailed (Figure 2C). In contrast, the AOC of IAC1246 started to increase at time point C and its fold change was maintained at much higher level through time points C to E (Figure 2C). The fold change of AOC was significant differences at time point B and D between IRAT109 and IAC1246 (Figure 2C). We recorded a significantly higher proportion of dead leaves in IRAT109 compared to IAC1246 (Table 1). As expected, the drought-tolerance coefficient was much lower in IRAT109 compared to IAC1246 (Table 1). All these results confirmed the contrasting drought-tolerances between both cultivars.

**Table 1:** **Proportion of dead leaf (mean ± SD), biomass (BIM, mean ± SD), and grain yield (GY, mean ± SD) measured in well-watered conditions (W) and under drought treatment (D)**.

**Cultivars**	**Proportion of dead leaves**	**BIM_W**	**BIM_D**	**GY_W**	**GY_D**	**Drought-tolerance coefficient**
IRAT109	0.468 ± 0.061^*^	15.97 ± 4.72^*^	7.90 ± 2.18^*^	8.90 ± 2.82	1.84 ± 1.03^*^	0.21
IAC1246	0.241 ± 0.060^*^	28.35 ± 6.80^*^	19.64 ± 4.35^*^	10.65 ± 2.74	7.89 ± 1.77^*^	0.74

*The drought-tolerance coefficient was calculated via GY_D÷GY_W*.

* *indicates significant differences between both cultivars via independent t-test*.

**Figure 3:**
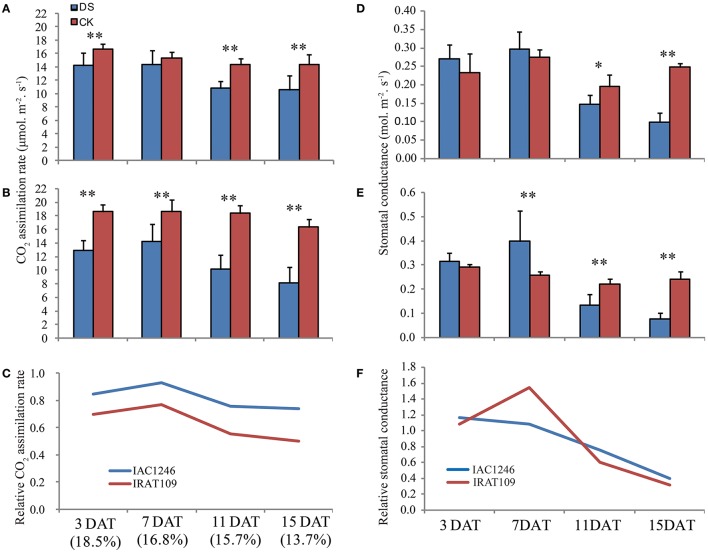
**Leaf gas exchange parameters measured during drought periods**. CO_2_ assimilation rate in IAC1246 **(A)**, IRAT109 **(B)**, and relative CO_2_ assimilation rate **(C)**. Stomatal conductance to water vapor in IAC1246 **(D)**, IRAT109 **(E)**, and relative stomatal conductance **(F)**. The relative value was calculated by value in drought stress (DS)/value in normal condition (CK). The bar indicates standard deviation. ^*^ and ^**^ indicates significant difference at *p* < 0.05 and *p* < 0.01, respectively. The percentages in brackets indicate soil water contents at these time points.

The photosynthesis rates of IAC1246 closely maintained normal levels at the early period of drought (3 and 7 DAT), while those of IRAT109 significantly decreased (Figures 3A,B). At the later drought (11 and 15 DAT), the photosynthesis rates of both IAC1246 and IRAT109 decreased significantly (Figures 3A,B). The relative photosynthesis rates (CO_2_ assimilation rate in drought/that in CK) of IAC1246 were much higher compared to those of IRAT109 throughout the whole drought period (Figure 3C). Furthermore, the stomatal conductance was only significantly decreased at a later period of drought for both cultivars (Figures 3D,E). Relative stomatal conductance (stomatal conductance in drought/stomatal conductance in CK) was almost similar in both cultivars (slightly higher in IRAT109) during the drought (Figure 3F). This result indicated the higher relative photosynthesis rate in IAC1246 to be independent of stomatal conductance.

### DEGs identified in IRAT109 and IAC1246

The drought-tolerant cultivar IAC1246 possessed far less DEGs (ranging from 548 to 766) than the drought-susceptible cultivar IRAT109 (ranging from 916 to 1700) during the drought periods (Table 2). However, the number of DEGs was much higher in IAC1246 compared to IRAT109 after re-watering (Table 2). We only detected a limited number of common DEGs between IRAT109 and IAC1246 at the six time points (Table 2). It should also be pointed out that the proportion of common DEGs between different time points was also very low in both IRAT109 and IAC1246 (Table S5). The cluster analysis based on the Log_2_FC of total DEGs supports the division of the drought process into three periods via soil-water contents, in which time point C, D, and E were always clustered (Figure S2). Interestingly, the average absolute FC value of total DEGs in IAC1246 was significantly higher than that in IRAT109 at all three periods (Figure S3). The FPKM value of the validated gene was well correlated with its relative expression via qPCR (Figure S4).

**Table 2:** **Number of up- and down-regulated DEGs detected at six time points in both cultivars IRAT109 and IAC1246**.

**Cultivars**	**Category of DEG**	**Sampling time point**					
		**A**	**B**	**C**	**D**	**E**	**F**
IRAT109	Up-regulated	588	346	749	768	868	437
	Down-regulated	371	570	227	676	832	136
	Total	959	916	976	1444	1700	573
IAC1246	Up-regulated	482	385	371	461	525	393
	Down-regulated	207	163	311	274	241	404
	Total	689	548	682	735	766	797
Common	Up-regulated	47	50	161	163	168	84
	Down-regulated	27	35	22	48	44	10
	Total	74	85	183	211	212	94

### Material-and period-specific DEGs and their KEGG/GO enrichments

**Figure 4:**
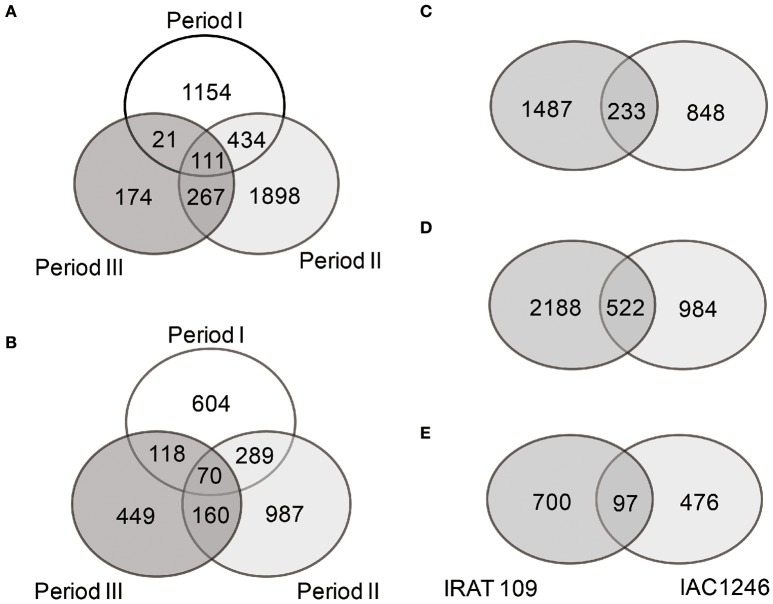
**Venn diagrams of DEGs among different drought periods in IRAT109 (A)** and IAC1246 **(B)** and between both cultivars at periods I **(C)**, II **(D)**, and III **(E)**, respectively.

**Figure 5:**
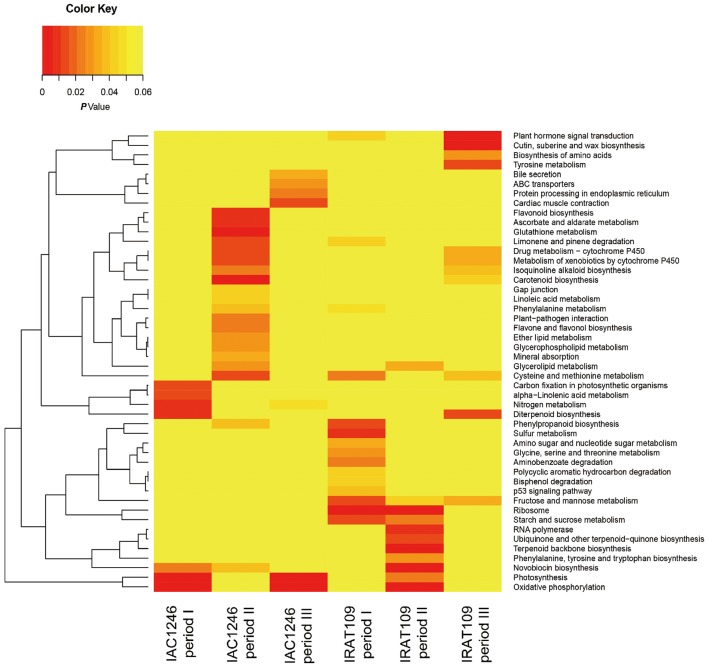
**Heatmap of enriched KEGG pathways at the three drought periods for IRAT109 and IAC1246**.

Totals of 4059 and 2677 DEGs were identified in IRAT109 and IAC1246, respectively (Figures 4A,B). For IRAT109, we found 1750, 2710, and 573 DEGs at periods I, II, and III, respectively, most of which were period-specific (Figure 4A). For IAC1246, we found 1081, 1506, and 797 DEGs, at periods I, II, and III, respectively, most of which were also period-specific (Figure 4B). In addition, when we compared DEGs from both cultivars for the same period, most of the DEGs were cultivar-specific (Figures 4C–E). The enrichment of KEGG pathways also revealed apparent period- and cultivar-specific patterns (Figure 5). Specifically, pathways of sugar and amino acid metabolism (e.g., starch and sucrose metabolism, fructose, and mannose metabolism, glycine, serine and threonine metabolism, amino sugar and nucleotide sugar metabolism) were preferably enriched in IRAT109. However, in IAC1246 pathways related to anti-oxidation activities (e.g., glutathione metabolism, flavone and flavonol biosynthesis, ascorbate and aldarate metabolism, and carotenoid biosynthesis) were enriched during the drought period (Figure 5). Notably, several KEGG pathways (e.g., carotenoid biosynthesis, metabolism of xenobiotics of cytochrome P450, drug metabolism-cytochrome P450, and isoquinoline alkaloid biosynthesis) that we found to be enriched in IAC1246 during period II were enriched in IRAT109 during period III (Figure 5). Similar to the enrichment of KEGG pathways, the enriched GO terms exhibited apparent cultivar- and period-specific results, suggesting different molecular mechanisms activated in these two cultivars (Table S6). For example, “water transmembrane transporter activity” and “water channel activity” were highly and uniquely enriched in IRAT109, while antioxidant related GO terms (e.g., hydroquinone, oxygen, oxidoreductase activity, and dioxygenase activity) were uniquely enriched in IAC1246 (Table S6).

**Figure 6:**
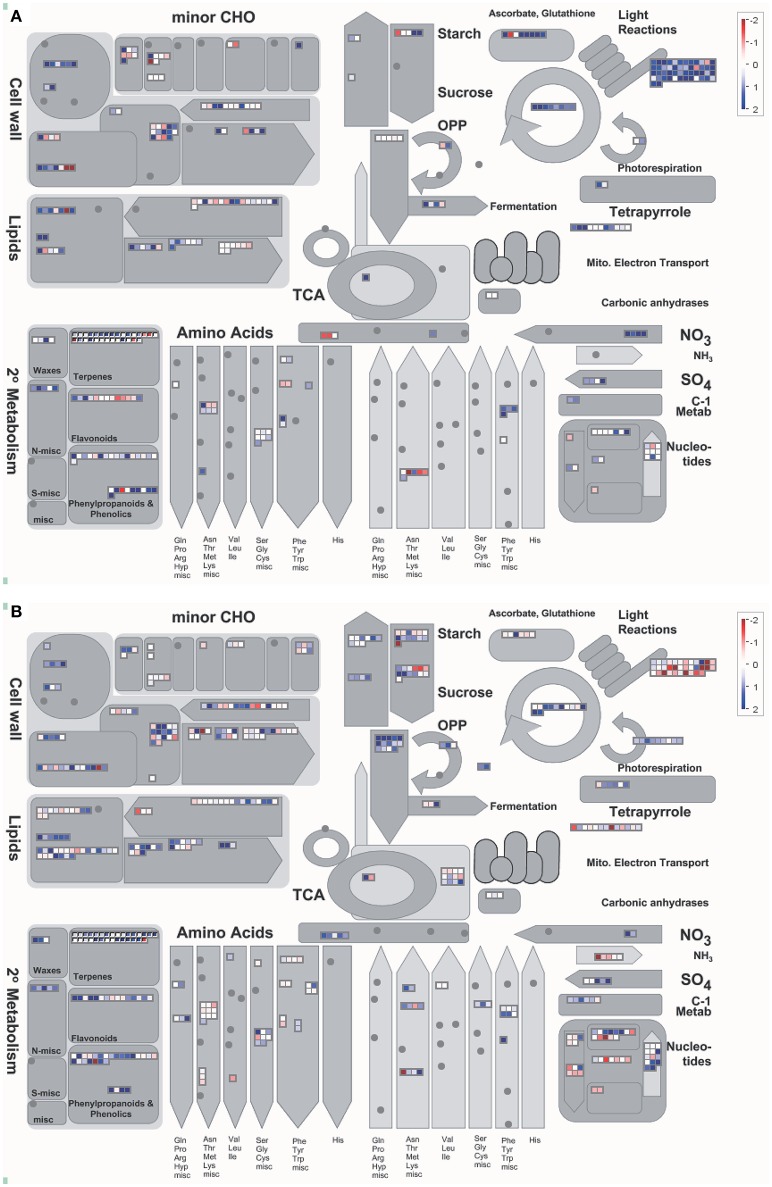
**Overview of the cellular metabolism analyzed via Mapman in IAC1246 (A)** and IRAT109 **(B)**. These values represent the median from log_2_(fpkm-D/fpkm-W) values of the five stages (A–E) for each DEG. Red and dark blue represent down-regulation and up-regulation, respectively.

Based on the Mapman results, the most apparent differences between IRAT109 and IAC1246 were the up-regulation of DEGs that are involved in the light reactions and metabolism of ascorbate and glutathione in IAC1246. However, the same DEGs were down-regulated in IRAT109 (Figure 6), particularly at time points A, B, and F (Figure S5). Additionally, more DEGs involved in the OPP cycle and in the metabolism of starch and sucrose were detected and up-regulated in IRAT109 (Figure 6).

### Regulations of osmolality-, AOC-, and photosynthesis-related DEGs in IRAT109 and IAC1246

**Figure 7:**
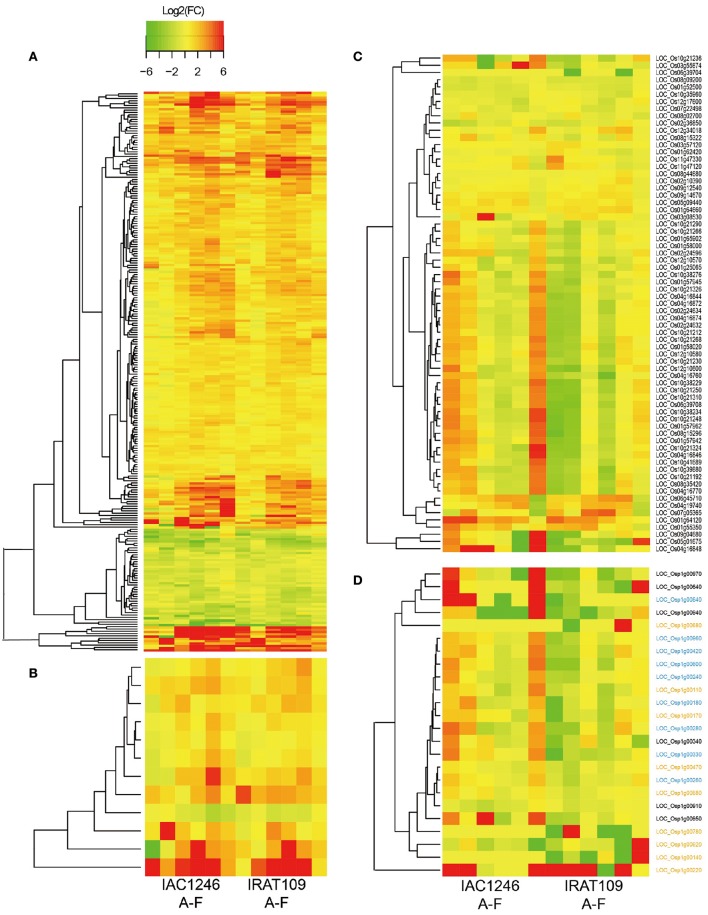
**Log_**2**_(fold-changes) of common osmolality-correlated DEGs in IRAT109 and IAC1246 (A)**, common AOC-correlated DEGs in IRAT109 and IAC1246 **(B)**, photosynthesis-related DEGs in IRAT109 or IAC1246 **(C)**, and chloroplast DEGs in IRAT109 or IAC1246 **(D)** at the six time points. If the value for log_2_ (fold-change) exceeded the range between −6 and 6, it was treated as −6 or 6 in the heatmap.

In IRAT109, we found 1200 DEGs (29.6% of total DEGs) and 268 (6.6% of total DEGs) significantly correlated with osmolality and AOC, respectively. 155 of these correlated with both osmolality and AOC (Table S7). In IAC1246, we found 772 (28.8% of total DEGs) and 293 (10.9% of total DEGs) DEGs significantly correlated respectively with the osmolality and AOC, 60 of these correlated with both the osmolality and AOC (Table S7). We found 241 common osmolality-correlated and 12 AOC-correlated DEGs that IRAT109 and IAC1246 shared (Figures 7A,B, Table S8). Although the general patterns of regulation of these common osmolality-correlated DEGs (Figure 7A, Table S8) and AOC-correlated DEGs (Figure 7B, Table S8) were similar in IRAT109 and IAC1246, we recorded higher amplitudes of their regulations in IAC1246 (Figures 7A,B).

Due to the general difference of light reactions between both cultivars indicated by the Mapman result, we paid particular attention to the regulation of photosynthesis related genes during the drought period. A total of 48 and 64 photosynthesis related genes were differentially expressed in IRAT109 and IAC1246. The photosynthesis-related DEGs displayed different regulatory patterns between IRAT109 and IAC1246, particularly at period I. Many of these DEGs were up-regulated in IAC1246, while they were down-regulated in IRAT109 (Figure 7C). The chloroplast genes in IRAT109 and IAC1246 exhibited a similar pattern of regulation compared to the photosynthesis-related DEGs (Figure 7D), providing additional evidence that the efficiency of photosynthesis differed between both cultivars.

### Differential metabolites detected in IRAT109 and IAC1246 during drought periods

**Figure 8:**
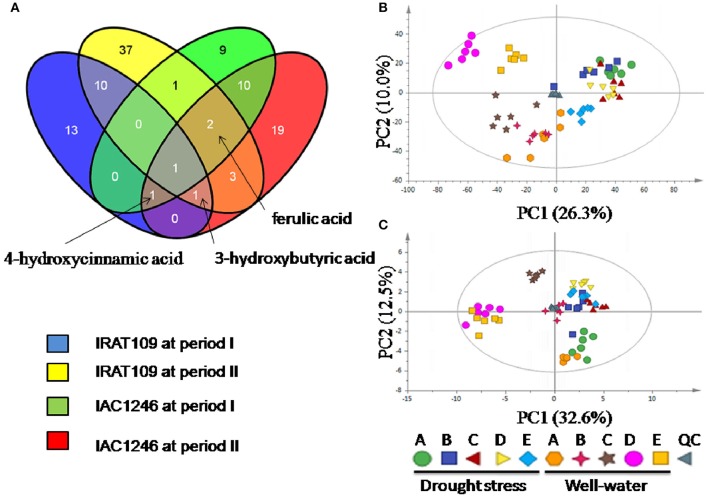
**Venn diagram of differential metabolites between drought periods and cultivars (A)** and principle component analysis (PCA) based on differential metabolites in IRAT109 **(B)** and IAC1246 **(C)**.

We detected a total of 185 and 130 metabolites in IRAT109 and IAC1246, respectively, 101 of which were shared between both cultivars. A total of 69 and 47 differential metabolites were identified in IRAT109 and IAC1246, respectively (Figure 8A). In IRAT109, we found 26 and 55 metabolites to be significantly different between the drought-stressed and well-watered conditions during periods I and II, respectively (Figure 8A, Table S9). In IAC1246, 24 and 37 metabolites had significant differences between the drought-stressed and well-watered conditions during periods I and II, respectively (Figure 8A, Table S9). Additionally, few differential metabolites were shared between these two rice cultivars (Figure 8A).

Depending on these differential metabolites, we were only able to separate samples from the drought-stressed and well-watered conditions *via* PCA analysis for IRAT109 (Figure 8B). However, for IAC1246, this was only possible for the drought-stressed samples from time points C to E of the well-watered condition (Figure 8C).

**Figure 9:**
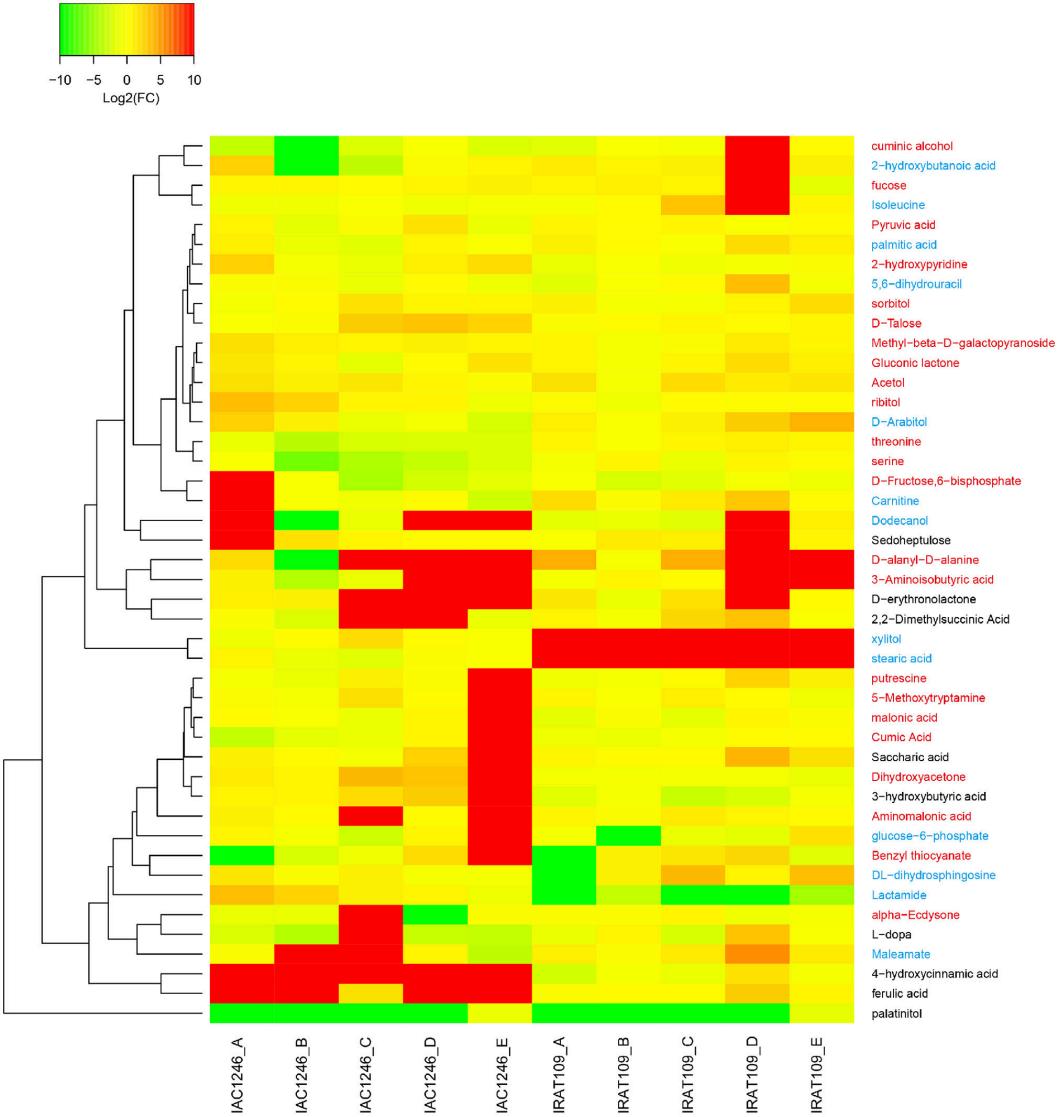
**Heatmap of fold-changes of differentials, detected in both IRAT109 and IAC1246**. If the value of log2 (fold-change) exceeded the range of −10 and 10, it was treated as −10 or 10 in the heatmap. We determined the metabolites in blue, red, and black on the right as differential metabolites in IRAT109, IAC1246, and both.

45 metabolites that were detectable in both IAC1246 and IRAT109 were determined as differential metabolites in IAC1246 and/or IRAT109 (Figure 9). Many of these metabolites represented different temporal patterns between IAC1246 and IRAT109 (Figure 9). For example, the contents of ferulic acid and 4-hydroxycinnamic acid were greatly increased in IAC1246 at drought period I, while they were only slightly increased in IRAT109 at the same period (Figure 9). Many differential metabolites were specifically up-regulated at time point E, including putrescine, 5-Methoxytryptamine, malonic acid, cumic acid, saccharic acid, dihydroxyacetone, 3-hydroxybutyric acid, aminomalonic acid, glucose-6-phosphate, and benzyl thiocyanate (Figure 9). However, we found increased contents of xylitol and stearic acid throughout the drought in IRAT109 while they had no apparent alterations in IAC1246 (Figure 9).

### Differential metabolites correlated with the osmolality, AOC and DEGs in IRAT109 and IAC1246

In IRAT109, we found 17 differential metabolites that significantly correlated with the osmolality and four metabolites that significantly correlated with AOC (Table S10). In IAC1246, we found 10 differential metabolites that significantly correlated with osmolality and one metabolite that significantly correlated AOC (Table S10). Among these osmolality and AOC-related metabolites, 10 metabolites were detected in both IRAT109 and IAC1246 (Table S10).

35–1013 DEGs were significantly correlated with 31 differential metabolites in IAC1246, while 47–1559 DEGs were significantly correlated with 22 differential metabolites in IRAT109 (Table S11). We paid particular attention to the 4-hydroxycinnamic acid and ferulic acid as these were up-regulated in IAC1246 at the period of early drought. 448 and 658 DEGs correlated with 4-hydroxycinnamic acid in IAC1246 and IRAT109, including 0 and 17 AOC-correlated, 347 and 157 osmolality-correlated, and 4 and 2 photosynthesis-related DEGs, respectively (Table S12). 1013 and 301 DEGs correlated with ferulic acid in IAC1246 and IRAT109, including 234 and 29 AOC-correlated, 599 and 17 osmolality-correlated, and 5 and 0 photosynthesis-related DEGs, respectively (Table S12). Some of the DEGs correlated with 4-hydroxycindnamic acid and ferulic acid anticipated their own metabolic pathway (Tables S13, S14).

### Metabolite-based method to explore candidate DEGs for drought-tolerance

Among the 10 DMs detected in both IRAT109 and IAC1246 that significantly correlated with osmolality or AOC (Table S10), six metabolites (D-Arabitol, Lactamide, palatinitol, 4-hydroxycinnamic acid, Aminomalonic acid, and ferulic acid) represent different regulations between IAC1246 and IRAT109 (Figure 9). In particular, 4-hydroxycinnamic acid and ferulic acid were determined as DMs in both IAC1246 and IRAT109. They exhibit apparent up-regulation during early drought in IAC1246, in accordance with the up-regulation of photosynthesis-related DEGs. Additionally, many osmolality- and AOC-correlated DEGs were correlated with these, as well as photosynthesis-related DEGs. These two metabolites referred to four KEGG pathways (Ubiquinone and other terpenoid-quinone biosynthesis, Tyrosine metabolism, Phenylalanine metabolism, and Phenylpropanoid biosynthesis), which were also supplemented by the transcriptome data (Figure 5, Table 3). Twelve osmolality- and/or AOC-correlated DEGs were involved in these four pathways (Table 3). As expected, these DEGs also correlated with both metabolites. Consequently, these DEGs are valuable potential targets for improving drought-tolerance, since they likely regulate the metabolism of 4-hydroxycinnamic acid and ferulic acid.

**Table 3:** **Candidate DEGs involved in KEGG pathways that are related to and correlated with 4-hydroxycinnamic acid and ferulic acid**.

**KEGG pathway**	**Metabolite**	**Id**	**DEGs in IAC1246**	**FC**	**Annotation**
Ubiquinone and other terpenoid-quinone biosynthesis	C00811	Osa00130	**LOC_Os01g60450**^†^	13.348	cytochrome P450, expressed
			**LOC_Os06g23684**^†^	1.862	*OsNAAT4*
			**LOC_Os11g42510**^†^	1.19	tyrosine aminotransferase, putative, expressed
Tyrosine metabolism	C00811	Osa00350	**LOC_Os06g23684**^†^	1.862	*OsNAAT4*
			**LOC_Os11g42510**^†^	1.19	tyrosine aminotransferase, putative, expressed
Phenylalanine metabolism	C00811	Osa00360	**LOC_Os01g60450**^†^	13.348	cytochrome P450, expressed
			**LOC_Os06g23684**^†^	1.862	*OsNAAT4*
			**LOC_Os11g42510**^†^	1.19	tyrosine aminotransferase, putative, expressed
Phenylpropanoid biosynthesis	C00811 C1494	Osa00940	LOC_Os01g32364^†^	2.796	Os1bglu1- beta-mannosidase/glucosidase homolog, expressed
			LOC_Os01g40870^†^	−1.566	*OsALDH2C1*
			**LOC_Os01g60450**^†^	13.348	cytochrome P450, expressed
			LOC_Os01g36240^*^	10.504	peroxidase precursor, putative, expressed

*The differential metabolites were differentially regulated between IAC1246 and IRAT109. FC indicates highest Log_2_ (fold change) during drought periods*.

† *indicates osmolality-correlated DEGs*.

* *indicates AOC-correlated DEGs. Gene ID in bold indicates involvement of the gene in multiple pathways*.

## Discussion

### Contrasting drought tolerances of both cultivars and experimental design provides reveal the underlying molecular basis of drought-tolerance

Drought-avoidance and drought-tolerance play different roles in plant resistant to drought stress. (Bernier et al., 2008; Luo, 2010; Parent et al., 2010; Hu and Xiong, 2014). It is therefore essential to separate drought-tolerance from drought-avoidance when studying its molecular basis. However, many previous studies combined or mixed both mechanisms, which may lead to a bias in the result (Wang et al., 2011; Witt et al., 2012). In this study, the soil layers of limited depth could well neutralize the capacities of root accessing water at different depth between both cultivars. The experiment in our modified field condition was equivalent to that in pots. Additionally, planting cultivars in the modified field created a condition of even soil water content as revealed by its coefficient of variance.

In this study, contrasting drought tolerances were detected between IRAT109 (drought-intolerant genotype) and IAC1246 (drought-tolerant genotype) under drought stress condition. Although IRAT109 has been considered as a drought-resistant cultivar and is widely used in rice breeding and QTL mapping for its drought-resistance (Luo, 2010; Lou et al., 2015), it possesses poor drought-tolerance. However, higher drought-tolerance was detected in IAC1246 due to its higher capacities of osmotic adjustment and antioxidant regulation during drought periods. In summary, a specifically designed experimental system and two typical rice cultivars of contrasting drought-tolerances offer good opportunities to study the underlying molecular basis of drought-tolerance via transcriptomic and metabolomic analysis.

### Different transcriptomic and metabolomic bases of osmotic adjustments and antioxidant capacities in drought-susceptible and drought-tolerant cultivars

For this study, large numbers of detected DEGs and DMs were cultivar- and period-specific. Only a small proportion of DEGs related to osmotic adjustment (13.9%) and antioxidant (2.2%) were shared by both cultivars, as well as by differential metabolites. All these results indicate different molecular bases to be involved in both rice cultivars of contrasting drought-tolerances, although the osmotic adjustment and the antioxidant regulation are the common drought-tolerance mechanisms.

The enrichments KEGG pathways based on the DEGs confirmed divergent molecular bases of drought-tolerances in the two rice cultivars, particularly on the osmotic adjustment. IRAT109 tends to activate pathways of the sugar metabolism while IAC1246 prefers pathways of lipid metabolism for their osmotic adjustment during the drought stress. These results are partially supported by the varying differential metabolites related to osmotic adjustment that was detected in IRAT109 and IAC1246. However, further confirmation it is required to analyse whether lipids have higher efficiency in osmotic adjustment compared to sugar molecules.

IAC1246 had almost twice the antioxidant capacity correlated DEGs compared to IRAT109. IAC1246 seemed to rely more on antioxidants and many biological processes like glutathione metabolism, ascorbate and aldarate metabolism, and flavone and flavonol biosynthesis were uniquely enriched in this cultivar. Furthermore, the fold changes of these DEGs were much higher in the drought-tolerant cultivar. These results indicate the distinct and highly regulated antioxidant system as a reasonable explanation for the increased drought-tolerance of IAC1246.

### Well-maintained photosynthesis contribute to improve drought-tolerance

The drought-resistance of a crop is determined by its relative biomass or yield during drought compared to that in normal conditions (Yue et al., 2006). Consequently, a well-maintained photosynthesis during drought should be a key requirement for drought-resistance. The mechanisms of drought-tolerance, including the osmotic adjustment and antioxidant, play an essential role in the maintenance of normal photosynthesis under drought-stress. Therefore, the capacity of photosynthesis under stress conditions should be a feature of drought-tolerance (Nounjan et al., 2016; Zhang et al., 2016). Additionally, positive correlations between osmotic adjustment and antioxidant capacity with photosynthesis have been reported in numerous previous studies (Heuer and Plaut, 1989; Shangguan et al., 1999; Ramachandra Reddy et al., 2004; Singh et al., 2015).

In this study, the rice cultivar of higher drought-tolerance possessed relatively higher biomass accumulation and grain yield under drought conditions. The expression of photosynthesis-related and chloroplast DEGs were up-regulated in IAC1246 at drought period I, while in IRAT109 they were down-regulated at the same time. This is consistent with the higher relative photosynthesis rate measured in IAC1246 compared to that of IRAT109. All these results represent an increased or well-maintained level of photosynthesis in the drought-tolerant cultivar, providing a reasonable explanation as to why IAC1246 produced relatively higher biomass and yield under drought stress. The superior maintenance of photosynthesis in IAC1246 should result from improved osmotic adjustment and antioxidant in this tolerant cultivar. Although further experimental confirmation is required, we suspect elevated levels of photosynthesis during a period of mild drought should contribute to a higher drought-tolerance in rice.

### Metabolite-based candidate genes determination for drought-tolerance in rice

Drought-tolerance is typically a complicated trait since it comprises several different mechanisms and thousands of genes with minor effect (Luo, 2010; Hu and Xiong, 2014). It is thus very difficult to enhance the drought-tolerance in rice by regulating a single gene. Instead, DEGs which could cause systematic effects have been preferred to be selected and applied in drought-tolerance breeding (Luo, 2010; Hu and Xiong, 2014), such as transcript factors (Hu et al., 2006; Jeong et al., 2012; Fang et al., 2015; Xu et al., 2015) and protein kinases (Saijo et al., 2000; Ning et al., 2010). Similarly, the metabolism of a metabolite commonly results from interactions of many genes and pathways. Regulating a particular plant metabolite could cause systematic alterations and potentially enhance the drought-tolerance (Garg et al., 2002; Taji et al., 2002; Nuccio et al., 2015). Example trehalose has been shown to stabilize dehydrated enzymes, proteins, and lipid membranes efficiently, as well as protect biological structures from damage during desiccation. The transgenic rice/maize with a trehalose-6-phosphate phosphatase gene, which is directly involved in the biosynthesis of trehalose can generate higher amount of trehalose. The cultivar thus exhibits higher drought-tolerance due to a well-maintained photosynthesis during drought (Garg et al., 2002; Nuccio et al., 2015).

In this study, we investigated the metabolic responses of two rice cultivars, particularly those metabolites that are related to the enhanced expression of photosynthesis-related DEGs. The content of ferulic acid and 4-hydroxycinnamic acid were largely increased during drought period I in IAC1246 and were only slightly increased in IRAT109 during drought period I. Consequently, these two differential metabolites (ferulic acid and 4-hydroxycinnamic acid) should be closely associated with well-maintained photosynthesis capacities of IAC1246 under the mild drought period. We thus decided to develop the metabolite-centered method to mine for candidate genes. This means the DEGs that are involved in the metabolism pathways of these two key metabolites will be promising target genes for improving rice drought-tolerance.

Ferulic acid was reported as one of the most effective photo-protectors, providing protection to photosynthesis during drought stress (Hura et al., 2007) and participates in the pathway of “phenylpropanoid biosynthesis.” 4-hydroxycinnamic acid (also named p-Coumaric acid) is located upstream of ferulic acid, which may influence the biosynthesis of ferulic acid (Figure S6). It refers to four pathways, including ubiquinone and other pathways of the terpenoid-quinone biosynthesis, tyrosine metabolism, phenylalanine metabolism and phenylpropanoid biosynthesis. Although, biological functions of 4-hydroxycinnamic acid in relation to drought-tolerance have not been reported in previous studies, we suspect that this metabolite and DEGs involved in its related pathways could contribute to drought-tolerance in rice. They thus are good targets to enhance drought-tolerance similar to trehalose. Six DEGs related to these two metabolites were correlated with osmolality or AOC. Two among these (LOC_Os01g40870 and LOC_Os01g60450) were further considered as candidate genes. LOC_Os01g40870 encodes an aldehyde dehydrogenase and participates in the reaction of ferulic acid biosynthesis from Coniferyl-aldehyde (Figure S6). Many other aldehyde dehydrogenase genes have been reported to be related to abiotic-stress tolerance in *Arabidopsis thaliana* by protecting the plants against lipid peroxidation and oxidative stress (Kotchoni et al., 2006; Chen et al., 2015). LOC_Os01g60450 locates upstream of 4-hydroxycinnamic acid and directly participates in its biosynthesis. This gene is highly up-regulated in IAC1246, which may lead to the increased content of 4-hydroxycinnamic acid in this cultivar. We believe these two genes to be promising candidates to improve rice drought-tolerance.

## Conclusion

Drought-tolerance, a vital part of drought-resistance, should be separated from drought-avoidance using meticulous scientific experimental designs. In this study, transcript and metabolic responses of two rice cultivars expressing contrasting drought-tolerances were evaluated under long-term drought stress in a well-controlled experimental field. They represented different osmotic adjustments and antioxidant capacities, resulting in different regulations of photosynthesis during progressive drought. Due to elevated relative photosynthetic rate during the early period of the drought, IAC1246 could accumulate more biomass and produce higher yield, resulting in drought-tolerance. As the photosynthesis capacity during drought is associated with osmotic adjustment and AOC, it could largely determine the drought-tolerance, different from drought-avoidance to maintain the water status of a plant. DEGs and differential metabolites contributing to well-maintained photosynthesis during drought are promising candidates for the improvement of drought tolerance. We found two metabolites (ferulic acid and 4-hydroxycinnamic acid) to be correlated with osmotic potential and/or AOC and differently regulated in these two cultivars. They should be thus associated with increased or well-maintained photosynthetic rates in the drought-tolerant cultivar *via* osmotic adjustment and/or antioxidant mechanisms. Based on these findings, two genes that directly precede the biosynthesis of the detected metabolites were considered as candidate DEGs, as they could introduce comprehensive and systemic effects in plant resistant to drought by protecting photosynthetic capacity. However, the metabolic responses accompanied by transcript responses to the progressive drought stress should be further studied in more rice genotypes to further detect key metabolites, pathways, and genes for improving rice drought-resistance.

## Author contributions

LL, XM, HX, HL, and LC designed this study. XM, HX, YL, XZ, and CS performed the experiments. XM, HX, and HW analyzed the data. XM, HX, and LL drafted the manuscript.

## Funding

This work was supported by grants from the National Program for Basic Research of China (2012CB114305), the Project of Subject Construction, the Shanghai Academy of Agricultural Sciences, 2015 (SAAS-2015(07), the National High-Tech Research and Development Program of China (2014AA10A603), the Project of Shanghai Talent Youth of Agriculture, 2015 (2121555), the Shanghai Municipal Commission of Science and Technology (15DZ2290700), the Shanghai Municipal Commission of Agriculture (2014-1), and the Shanghai Municipal Commission of Science and Technology (12JC1408000).

### Conflict of interest statement

The authors declare that the research was conducted in the absence of any commercial or financial relationships that could be construed as a potential conflict of interest.

## Availability of supporting data

The raw Illumina sequencing data of this study have been submitted to the NCBI Sequence Read Archive (SRA) under the accession number PRJNA306542.
