# Supplementary material for: Transcriptomic and Metabolomic Studies Disclose Key Metabolism Pathways Contributing to Well-maintained Photosynthesis under the Drought and the Consequent Drought-Tolerance in Rice
Source: Front Plant Sci. 2016 Dec 21;7:1886. doi: 10.3389/fpls.2016.01886 (PMC5174129; doi:10.3389/fpls.2016.01886)
Supplement: Supplementary file 1 [file Table1.DOC]

**Table S1.** Pre-evaluation* of drought-resistance and drought-tolerance in 2011. ‘W’ indicates the well-watered condition while ‘D’ indicates the drought-stressed condition.

| Materials | Subspecies | Experiment in field of 2011 | | | Experiment in buckets of 2011 | | |
| --- | --- | --- | --- | --- | --- | --- | --- |
|  |  | Grain Yield _W | Grain Yield _D | Drought-resistant index | Grain Yield _W | Grain Yield_D | Drought-tolerant coefficient |
| IAC1246 | *Japonica* | 16.57 | 24.68 | 1.32 | 28.20 | 21.89 | 0.78 |
| IRAT109 | *Japonica* | 8.14 | 10.34 | 1.12 | 15.78 | 3.15 | 0.20 |

***Procedures of pre-evaluations of the drought-resistance and drought-tolerance:**

To estimate the drought-resistance, two cultivars were grown in the fields of two water regimes using the drought resistance facility in 2011 at Baihe Experimental Station of Shanghai Agrobiological Gene Center (31^o^15′N, 121^o^10′E). The gradual water regimes were created *via* the drip irrigation along the sprinkler where the well-watered regime was near the sprinkler and drought regime was far away from the sprinkler . Each cultivar grew in two replicates with four rows (18cm interval) of 22 hills in each row (16cm interval). The drought treatment was started at the early booting stage and lasted for 35 days until the soil water content declined from 23.1% to 10.0%. Single factor randomized block design and two biological repetitions were applied in this study. The drought-resistant index was calculated following the methods described by Yu et al .

Meanwhile, to estimate their drought-tolerances, two cultivars grew in plastic buckets (30cm×20cm×40cm) with soil layers of 30cm depth in the same year at the Baihe Experimental Station. Each cultivar grew in six buckets and each bucket contained four rice seedlings. Equal amount of water was used in the experiment for those buckets until the drought treatment. Half of the six buckets were treated by drought and the other half remained well-watered (possessing a shallow layer of water in the bucket) as controls. The drought treatment was started from panicle initiation stage to the grain filling stage lasting for 30 days. The soil water content ranged from 8.5%-10% in these drought-stressed buckets before re-water. The soil water content ranged from 8.5%-10% in these drought-stressed buckets before re-water. As the thickness of the soil layer in bucket was only 30cm, the root development to the depth of these rice cultivars was largely limited and the abilities of water-uptake at depth by roots were neutralized among cultivars [3]. When the seeds matured both in control and drought treated buckets, they were harvested and their drought-tolerance coefficients were calculated as: yield under drought stress/yield under well-water conditions.

**Reference**

1. Luo LJ: Breeding for water-saving and drought-resistance rice (WDR) in China. *J Exp Bot* 2010, 61(13):3509-3517.
2. Yu S, Liao F, Wang F, Wen W, Li J, Mei H, Luo L, Identification of rice transcription factors associated with drought tolerance using the Ecotilling method, PLoS One 2012, 7: e30765.
3. Parent B, Suard B, Serraj R, Tardieu F: Rice leaf growth and water potential are resilient to evaporative demand and soil water deficit once the effects of root system are neutralized. *Plant Cell Environ* 2010, 33(8):1256-1267.
