# Supplementary figures and images for: Transcriptomic and Metabolomic Studies Disclose Key Metabolism Pathways Contributing to Well-maintained Photosynthesis under the Drought and the Consequent Drought-Tolerance in Rice
Source: Front Plant Sci. 2016 Dec 21;7:1886. doi: 10.3389/fpls.2016.01886 (PMC5174129; doi:10.3389/fpls.2016.01886)

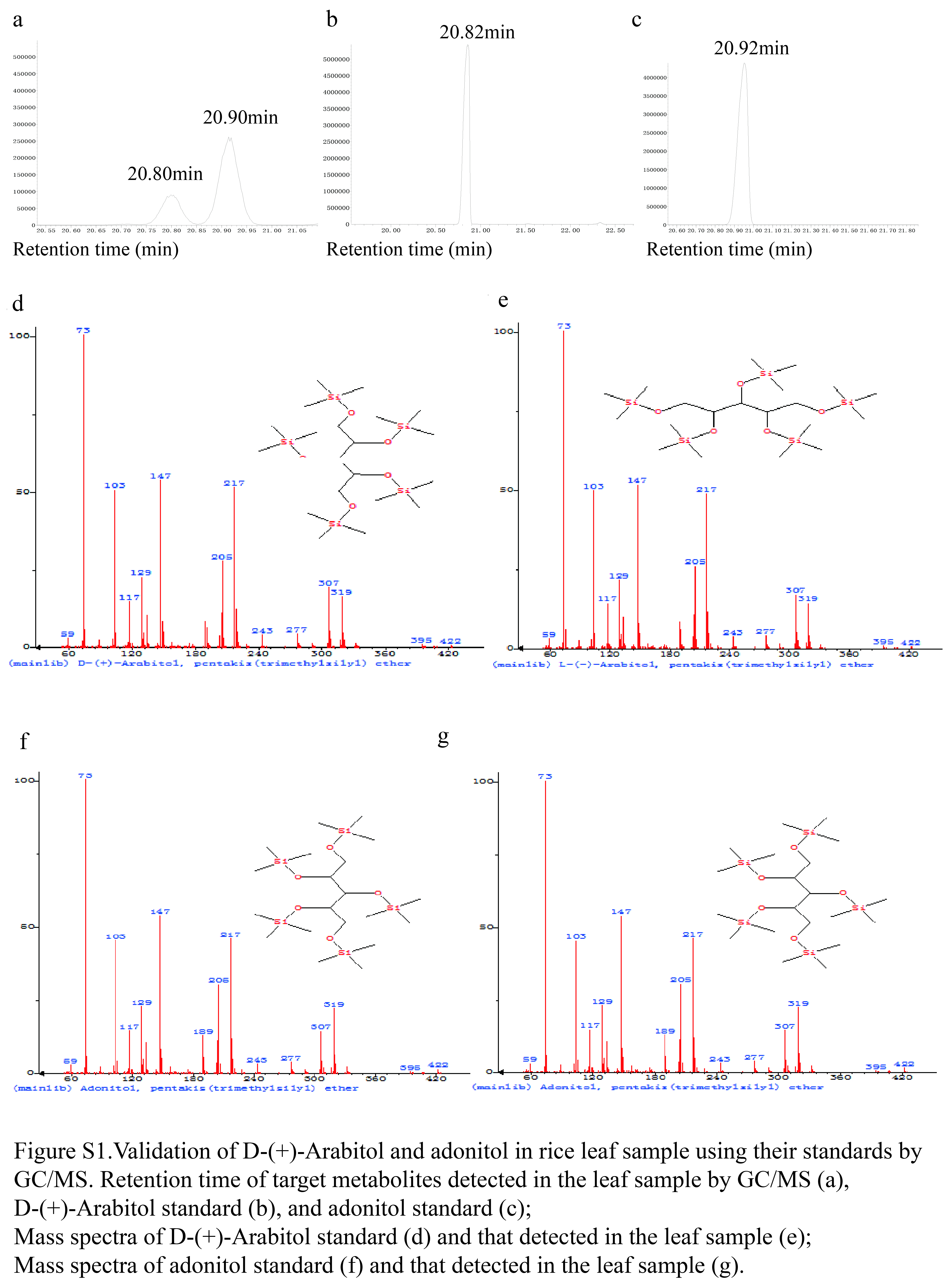

Supplement: Supplementary file 15 [file Image1.JPEG]

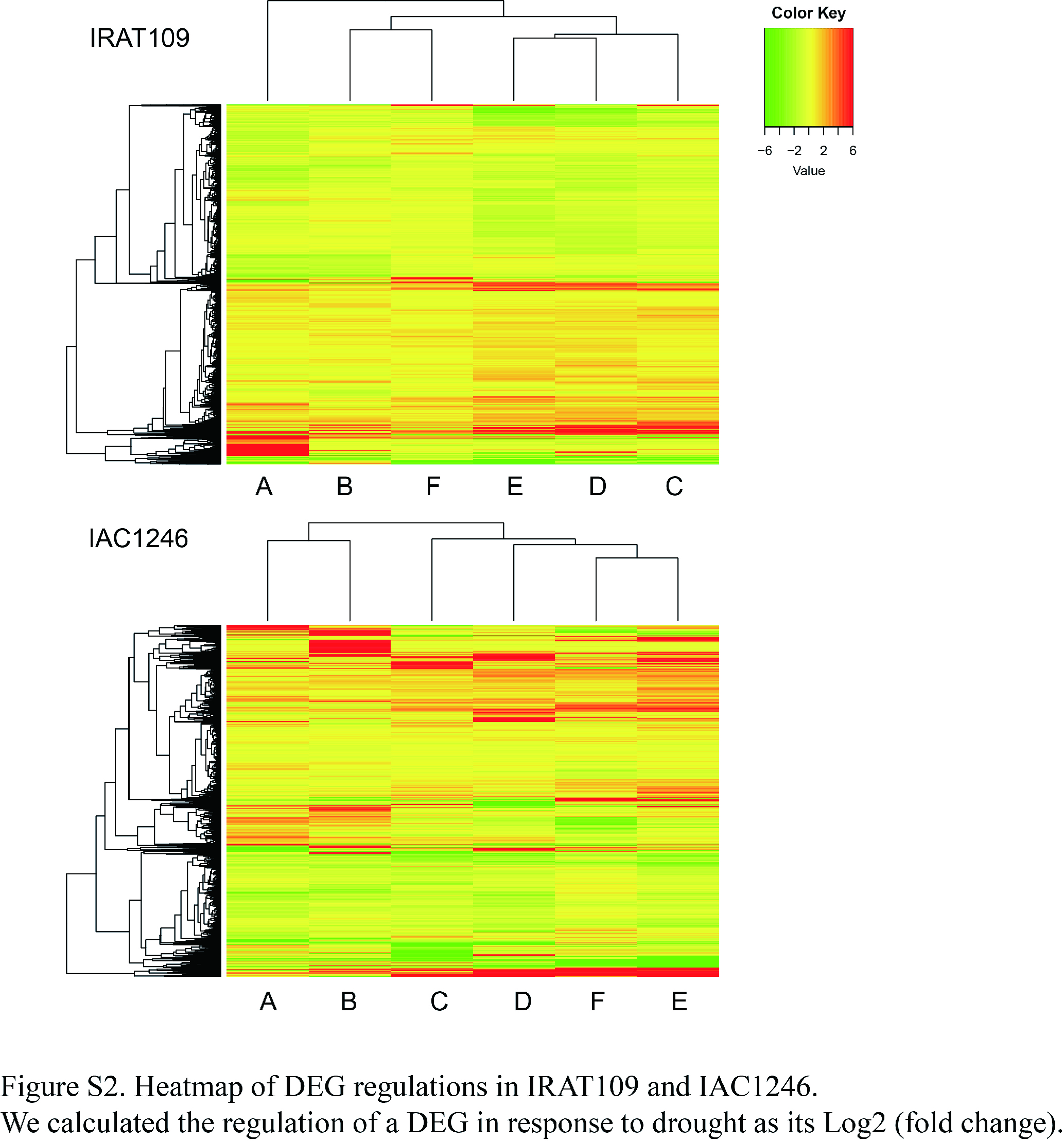

Supplement: Supplementary file 16 [file Image2.JPEG]

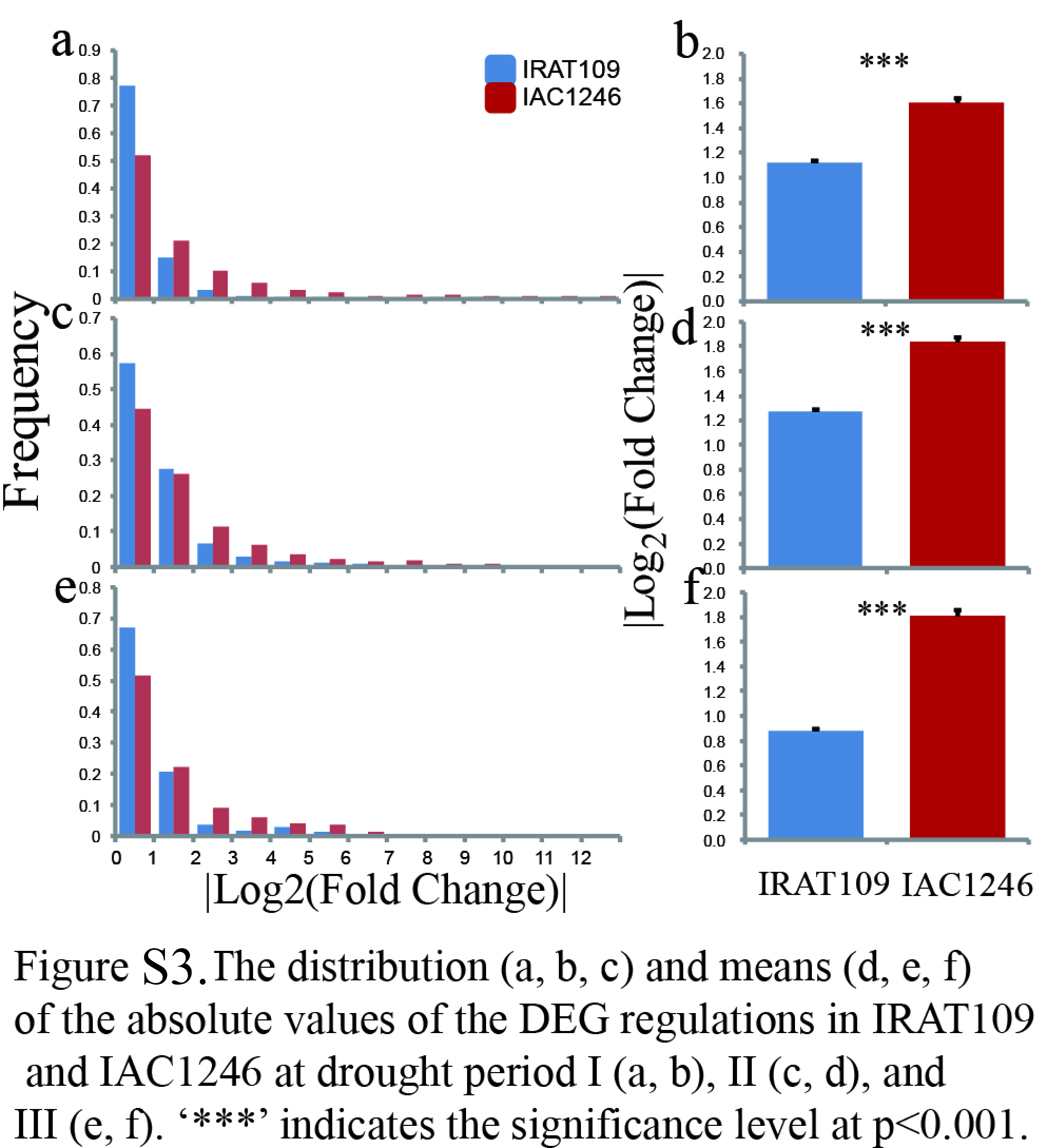

Supplement: Supplementary file 17 [file Image3.JPEG]

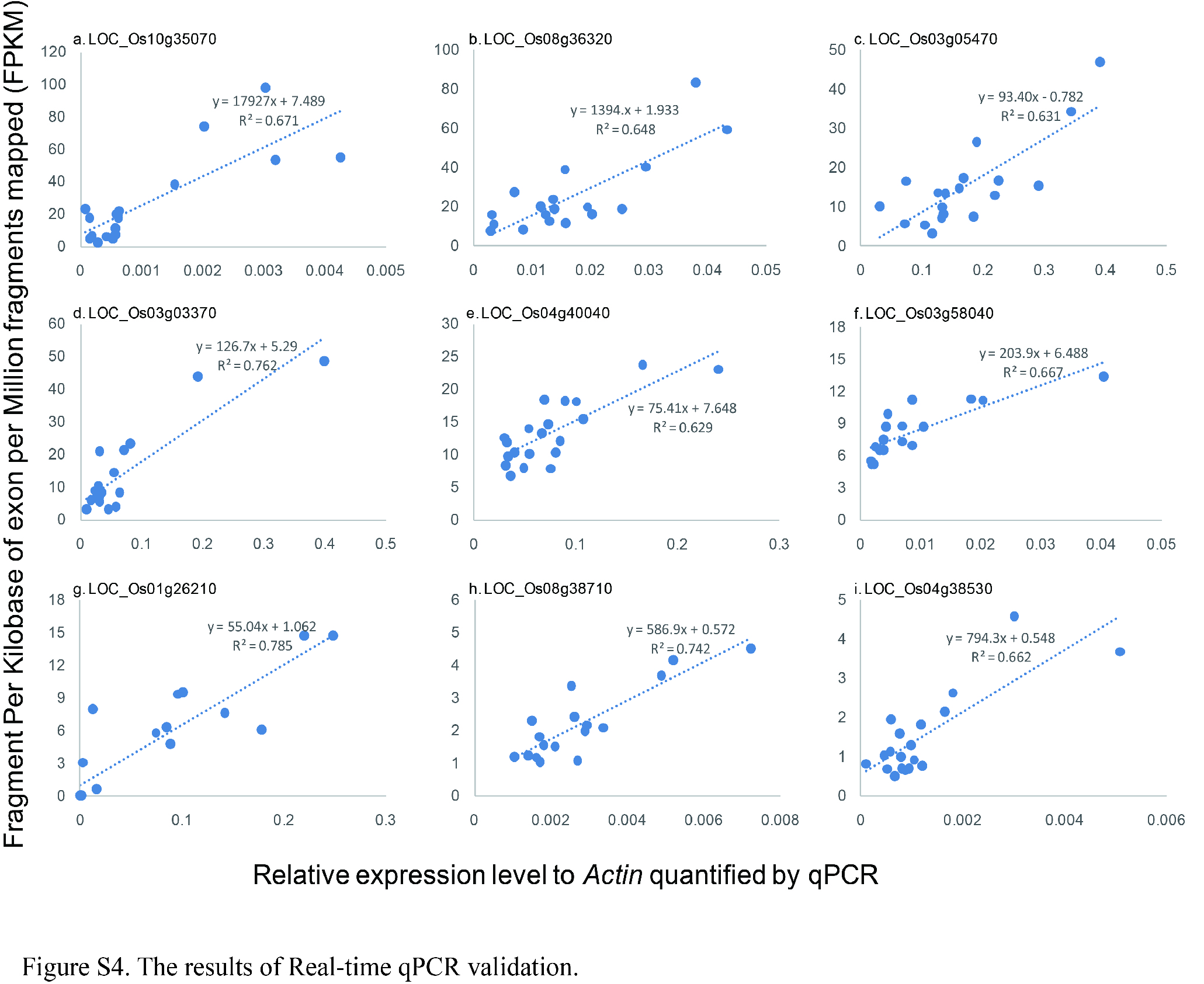

Supplement: Supplementary file 18 [file Image4.JPEG]

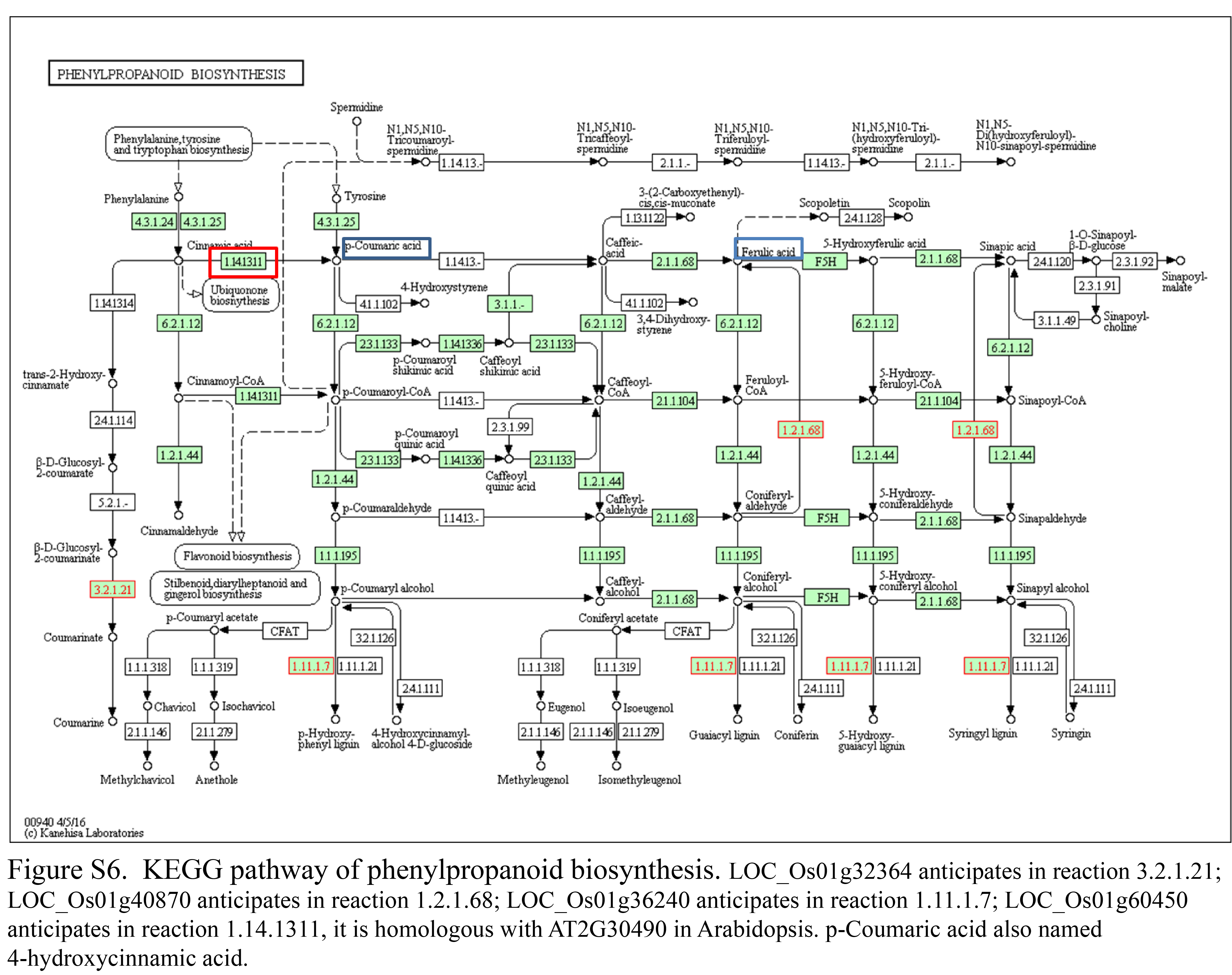

Supplement: Supplementary file 20 [file Image6.JPEG]
