# Supplementary material for: Transcriptomic and Metabolomic Studies Disclose Key Metabolism Pathways Contributing to Well-maintained Photosynthesis under the Drought and the Consequent Drought-Tolerance in Rice
Source: Front Plant Sci. 2016 Dec 21;7:1886. doi: 10.3389/fpls.2016.01886 (PMC5174129; doi:10.3389/fpls.2016.01886)
Supplement: Supplementary file 19 [file Image5.pdf]

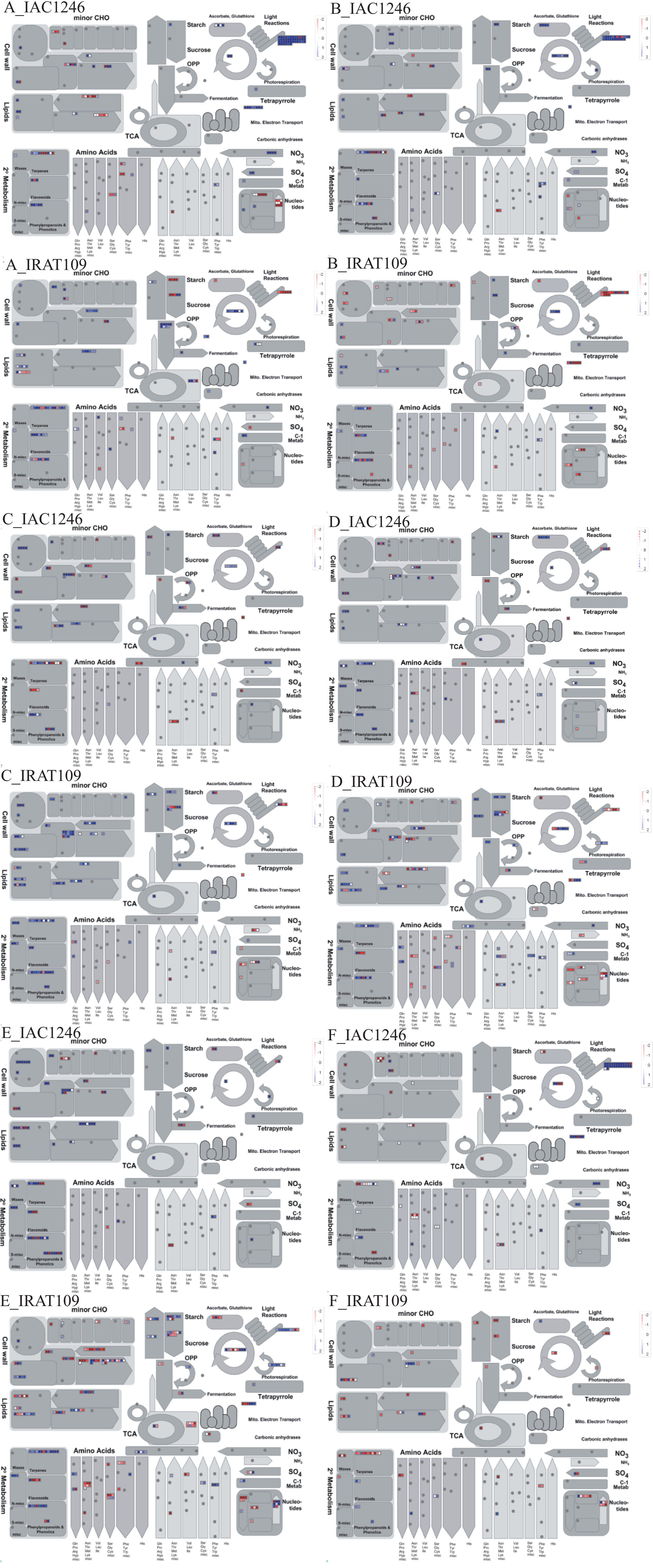

Figure S5. Overview of cellular metabolism analyzed by Mapman in IAC1246 and IRAT109. A, B, C, D, E, F represent from A to F time point, respectively. These values represent log2(fpkm-D/fpkm-W) values.
